# Supplementary material for: Are carbon emissions trading and green financial instruments synergistic? -Comprehensive quantitative research based on content analysis
Source: PLoS One. 2024 Mar 7;19(3):e0298601. doi: 10.1371/journal.pone.0298601 (PMC10919601; doi:10.1371/journal.pone.0298601)
Supplement: S1 Data — (ZIP) [file pone.0298601.s001.zip › Data/地级市Z_F工作报告.zip!/╣π╢1⁄2/╔╟╬▓╩╨/└┤╫╘╣1⁄2╓┌║┼í╛┬φ┐╦╩2╛▌═°í┐_2014.docx]

各位代表：

　　我代表市人民政府向大会作政府工作报告，请予审议，并请政协各位委员和其他列席人员提出意见。

**2013年工作回顾**

　　2013年是我市全面贯彻落实党的十八大和习近平总书记系列重要讲话精神的开局之年，在省委、省政府及市委的正确领导下，在市人大及其常委会和市政协的监督支持下，市政府坚持以邓小平理论、“三个代表”重要思想和科学发展观为指导，按照市委六届二次全会部署，凝心聚力，抢抓机遇，心无旁骛向西发展，更加主动融入珠三角，坚定不移加快发展，全力落实“四大行动”，顺利完成了市六届人大三次会议确定的各项任务。

　　（一）经济保持较快健康发展

　　2013年全市生产总值671.75亿元，增长12.2%；规模以上工业增加值234.98亿元,增长24.9%；固定资产投资总额462.09亿元，增长22.3%；公共财政预算收入48.15亿元，增长17.2%，财税质量有所提高，非税收入占36.95%，比上年降低近2个百分点；国税税收收入突破30亿元，地税税费收入突破50亿元；社会消费品零售总额473.56亿元，增长11.6%；外贸进出口总额41.7亿美元，增长46.9%；居民消费价格上升2.5%；市区居民人均可支配收入20804元，增长12.9%，农民人均纯收入9563元，增长11.6%；预计万元GDP能耗下降5.18%。

　　（二）转型升级步伐加快

　　——产业结构调整优化。工业发展加快，电子信息、电力能源、工艺品制造、纺织服装四大主导产业实现增加值156.82亿元，占规模以上工业增加值66.7%，对工业增长的贡献率达79%。商贸、旅游、金融等现代服务业加快发展。第三产业完成增加值247.81亿元，占GDP的36.9%；旅游总收入82.04亿元，增长13.2%；红海湾滨海旅游产业园区完成总体规划，项目工程加快推进；银行金融机构各项存款余额485.32亿元，增长14.8%，各项贷款余额228.54亿元，增长19.8%，存贷比提高2个百分点。

　　——重点项目进展顺利。省重点建设项目完成投资55.6亿元，占年度计划112%；市重点建设项目完成投资107亿元，占年度计划120%。厦深铁路于去年底正式通车，汕尾迎来了第一列高速火车。潮惠高速公路全线开工建设，管理中心建成投入使用。陆丰核电解决了征地赔偿等遗留问题，完成1号核岛负挖工程。海丰华润电厂1、2号机组进入设备安装和码头施工阶段。红海湾电厂5、6号机组扩容正在报批。陆丰甲湖湾电厂行政区全面开工建设。深能源汕尾滨海电厂完成了初步可行性研究。中电国际陆丰电厂和华润水泥等项目开展选址等前期工作。

　　——区域合作不断深入。深圳全面对口帮扶汕尾工作正式启动。与深圳、东莞、惠州等珠三角城市合作交流不断深入。深汕特别合作区起步区加快推进,腾讯云计算数据中心完成投资1.4亿元，瑞和产业园建设加快，华润大数据中心项目正在选址。

　　——产业园区加快建设。汕尾红草产业转移工业园被省认定为“省市共建循环经济产业基地”。海丰县产业转移工业园1.2平方公里起步区加紧推进，30多家企业落户投建。陆丰市产业转移工业园正在进行土地清理及调整；陆河县产业转移工业园已投入3.8亿元，“七通一平”工作加快推进。

　　——农业产业稳步发展。农业增加值108.26亿元，增长3.9%；粮食总产量41.72万吨。农业产业化不断推进，粮食、无公害农产品、特色农产品等生产基地初具规模，全市拥有各级农业龙头企业64家，其中国家级1家、省级10家；新增省级菜篮子基地16个，培育基地9个。投入4.4亿元完成中央和省级民生水利项目125宗，改善农田灌溉面积1.1万亩。及时启动政策性水稻保险工作，全年赔付受灾农户900万元。全国500强企业浙江海亮集团落户海丰县发展有机农业。鲘门、小漠标准化渔港加快建设。

　　——扩容提质扎实推进。465.1平方公里的汕尾新区规划获省批准。中心城区扩容提质加快推进，全市综合交通规划、市区火车站片区控规、红草产业转移工业园控规等规划编制工作全面完成；环品清湖片区、火车站站前广场等市政配套设施加快建设。碧桂园二期建设推进顺利。《汕尾市土地利用总体规划调整方案》获省批准，全市306个重点项目列入规划，为项目落地提供规划支撑；市区新储备土地3000亩，火车站、金町湾滨海旅游区等片区征地工作取得较大进展；市区土地清查工作成效明显，已收回土地48宗43.89公顷。启动省卫生城市创建活动。完成环卫保洁市场化改革。

　　——企业不断发展壮大。全市规模以上工业企业实现利税总额52.45亿元，增长28.8%；实现利润总额35.95亿元，增长38.9%。新增个体工商户9618户、私营企业713家。我市获得全国科技进步考核先进市。创办4家研发机构、7家企业研发中心；专利申请1176件，增长55%；专利授权818件，增长69%；成功申请省著名商标3个，新增省名牌产品2个；维明科技公司“海藻精深加工技术”获省科学技术一等奖；信利光电公司“液晶显示装置制作方法”获省专利优秀奖。

　　——外向经济加快转型。进出口总值突破40亿美元，增长46.9%。高新技术产品出口约占关区5市（包括粤东四市和梅州市）出口总量的60%。加工贸易“委托设计+自主品牌”生产方式出口比重达60%。专业化招商全面铺开，省属企业投资对接加快推进，金町湾项目、鲘门观海项目等一批重点项目推进顺利。

　　——重点领域改革不断深化。行政审批制度改革扎实推进，调整行政审批事项207项，市县行政审批改革事项第一批目录全部公布。网上办事大厅建成开通，可受理1091项政务服务事项。政务服务中心正式运作。市食品药品监管体制改革、卫生和计生、新闻出版和广电部门机构改革，公共资源交易体制改革顺利实施，职能有效整合。事业单位分类改革基本完成。公立医院及相关领域改革加快推进，中信医疗健康产业项目已签订框架协议。信利光电等一批重点企业上市前期工作力度加大。农村金融改革有效推进，我市首家股份制地方金融机构汕尾农商行于今年1月26日挂牌开业。

　　（三）民生福祉不断改善

　　——社会保障能力不断增强。全市城镇新增就业45503人，城镇登记失业率2.44%；农村劳动力转移就业率75%；参加城镇职工基本养老保险45万人（含企业退休人员4.63万人），参加城乡居民养老保险90万人，其中60周岁以上领取养老待遇29万人，均100%完成年度任务。参加城乡三项基本医疗保险293万人，参保覆盖率达98.7%。社会救助体系逐步完善，社会福利和慈善事业稳步发展。扶贫工作在中山市的大力帮扶下，全面实现三年目标任务，受到省委、省政府的通报表扬；新一轮深圳市帮扶我市90个村5804户贫困户，已到位帮扶资金2.7亿元，实施帮扶项目1020个。

　　——社会事业加快发展。公共文化教育服务体系不断完善，全面完成“广播电视渔船通”、“户户通”工程建设。“欢乐广场?唱响汕尾”群众文化活动被评为省群众性文化活动优秀品牌。陆丰金属雕、甲子贝雕、碣石木雕入选第五批省级非物质文化遗产名录。完成县级软件正版化工作任务。教育创强工作稳步推进，16个创强镇向省申报验收；高考取得历年最好成绩，一本上线人数增长34.2%，本科上线人数增长11%；高中阶段毛入学率86.9%；投入1.8亿元的教育信息化加快建设，今年新学期可实现全市公办中小学课室多媒体电教平台全覆盖。医疗保障大幅提升，基层医疗卫生服务体系进一步完善。幸福村居创建成效明显，掀起了村头公园建设热潮。计生殡改工作取得新成效；体育、物价、统计、监察、审计、侨务、档案、修志、国防、人防、口岸、港澳台、民族宗教、残联、工青妇等工作取得新成绩。

　　——十件民生实事基本完成。逸挥基金医院肿瘤防治中心建设加快推进，市人民医院综合大楼配套进一步完善；城乡基本医疗保险筹资标准由240元提高到280元；建成公租房799套；完成135公里农村公路硬底化建设；投入1880万元建设以市区为重点的景观林带；投入1760万元建设完善11个镇（街道）综合文化站、240个村（社区）文化室，建成镇村电子阅览室62个；全省首个地震预警台在我市开工建设；环境空气监测网建设基本完成。

　　——应对灾害有为有效。全力以赴做好“8?16”洪涝抗灾工作，紧急转移安置群众5.2万人。强台风“天兔”正面袭击我市，全市63个乡镇、225万人受灾，房屋倒塌3167间，直接经济损失巨大。面对我市有史以来的最强台风，全市人民万众一心，众志成城，紧急转移安置群众25万人，及时出台扶持复产重建政策，抗灾复产重建家园工作有效开展，春节前因灾的“全倒户”全部入住新居。

　　——平安建设有效推进。公共安全体系不断健全，突发事件应对处置水平不断提高。完善信访排查机制，畅通信访渠道，有效化解信访突出问题，妥善处置城区东涌土地问题，到省、市上访和信访人数批次全面下降。食品药品安全、安全生产形势平稳。社会治安综合治理逐步加强，重点地区突出问题有效整治，深入开展“雷霆扫毒”行动，清剿涉毒严重的陆丰市博社村，摧毁18个制贩毒犯罪团伙，抓捕嫌疑人182名，缴获冰毒近3吨、制毒原料23吨，扭转了陆丰禁毒维稳工作被动局面；海防打私工作成效明显；立体化治安防控体系建设加快推进，社会大局总体稳定。

　　过去一年，我们坚持加强政府自身建设，不断完善决策机制，提高依法行政水平。坚持向人大及其常委会报告工作，向人民政协通报情况，自觉接受监督，办理省、市人大代表议案建议27件、政协提案102件。认真听取各民主党派、工会、共青团、妇联等群团意见。严格落实中央“八项规定”和省实施办法，以改进机关作风、优化政务环境为目标，扎实开展政务整治行动，政府廉政建设取得新进展，机关精神面貌明显改善，服务效能明显增强，发展环境明显优化。

　　各位代表！过去一年我市取得的成绩来之不易，饱含着省委、省政府的深切关怀，归功于市委的坚强领导和市人大、市政协的监督帮助，凝聚了全市人民的智慧汗水，得到了海内外各界人士的关心支持。在此，我代表市人民政府，向广大市民和各界人士表示崇高的敬意！向长期关心支持我市经济社会发展的港澳台同胞、海外侨胞表示衷心的感谢！

　　我们也清醒地看到，我市经济社会发展还存在不少问题和困难：一是经济总量小，产业基础弱，企业规模小，经济效益差的状况仍未得到根本改变，去年财政非税收入比重虽有明显下降，但还比较高，发展质量和水平有待进一步提高。二是城市公共服务水平较低，供给能力弱，教育、医疗、住房、城市建设、社会保障等民生事业欠账多，三甲医院、博物馆、文化馆、体育场等应有未有，与人民群众的需求差距较大；城镇化建设滞后，中心城区辐射带动作用不强。三是社会管理水平不高，交通秩序、市容市貌、生态环境有待进一步改善，毒品问题仍需进一步整治，影响社会和谐稳定因素较多，社会治安压力大、任务重。四是作风建设有待进一步加强，干部抓落实的拼劲和韧劲不足，政府服务和办事效率与上级和人民群众的要求还有很大差距。对此，我们必须高度重视，采取有2014年工作安排

　　2014年是全面贯彻落实党的十八届三中全会精神、全面深化改革的第一年，是完成“十二五”规划目标任务的关键一年。当前我市正处于深化改革、转型升级、加快发展的黄金时期。随着中央关于全面深化改革战略部署的推进，省委、省政府《关于进一步促进粤东西北地区振兴发展的决定》的深入实施，深圳对口帮扶汕尾工作的全面启动，高铁经济时代的到来，我市实现振兴发展正迎来难得的机遇。做好今年工作，我们必须坚定信心，提振精神，坚持改革创新、加快发展不动摇。既要巩固质量向好的发展态势，促进经济社会大局稳定，为改革创造必要条件，又要以经济体制改革为总领，全面推进各领域改革，努力在全面深化改革中推动全市经济社会加快发展。

　　2014年政府工作的总体要求是：全面贯彻落实党的十八大、十八届三中全会、中央经济工作会议和省委十一届三次全会、全省进一步促进粤东西北地区振兴发展工作会议精神，深入贯彻习近平总书记系列重要讲话和省委胡春华书记关于汕尾工作的重要指示精神，按照市委六届三次全会的部署，把改革创新贯穿于经济社会发展各个领域各个环节，抓住深圳多领域全方位对口帮扶汕尾的契机，全面融入珠三角，深入开展“平安建设、招商引资、政务整治、形象提升”四大行动，加快转型升级，着力改善民生，坚定不移加快发展，坚定不移提高经济发展质量和效益，努力建设成为全省滨海旅游集聚区、宜居宜业宜游现代化滨海城市、珠三角产业拓展集聚地，为实现与全国同步全面建成小康社会总目标而努力奋斗。

　　2014年的主要预期目标是：地区生产总值增长14.5%；人均GDP增长13.5%；规模以上工业增加值增长27%；固定资产投资增长30%；社会消费品零售总额增长13%；外贸进出口总额增长18%；公共财政预算收入增长16%（其中税收收入增长20%，非税收入比重下降2个百分点）；城镇居民人均可支配收入增长13%；农村居民人均纯收入增长12%；居民消费价格涨幅控制在3.5%左右；城镇登记失业率控制在2.5%以内；节能减排约束性指标完成省下达的年度计划。为此，必须着力抓好八项工作：

　　（一）加快重点项目建设，推动产业优化升级

　　坚持狠抓重点项目不动摇，以项目促转型、增后劲，全力推进产业转型升级，不断增强发展内生动力，促进经济加快发展。

　　——大力推进重点项目建设。今年安排市重点建设项目48项，总投资1163亿元，年度计划投资134亿元。加快建设陆丰核电、华润海丰电厂、陆丰甲湖湾电厂等电力能源项目，信利光电、腾讯云计算等电子信息项目，城市综合体、金町湾等商贸旅游项目，力争陆丰核电和甲湖湾电厂获国家正式核准并动工建设，确保华润海丰电厂1台机组建成投产。

　　——大力发展旅游业。以厦深铁路通车为契机，进一步完善旅游规划，建设旅游度假胜地。创新投融资体制，充分发挥省竞争性扶持资金的放大效应，加快红海湾滨海旅游产业示范园区、鲘门滨海度假区、碣石玄武山——金厢滩宗教滨海旅游区、海丰湿地鸟类休闲旅游区、赤石明热温泉度假区等项目建设。启动环品清湖国家级海洋公园和国家级5A景区的规划建设工作。

　　——优化提升商贸流通业。加快火车站国际商贸物流城、粤东水产物流中心、家具广场等项目建设，打造一批高端商贸综合体。扶持企业开展电子商务营销，引导社会资本建设汕尾产品电子交易平台，促进电子商务服务业规模化、产业化、规范化发展。推进“万村千乡”市场改造升级、农超对接和平价商场建设，培育农村流通合作服务组织。

　　——大力推动传统产业改造升级。实施技术标准带动战略，抓住深圳市对口帮扶机遇，大力实施传统产业提升计划，支持传统产业加快向产品研发、设计、营销等附加值高的产业环节延伸，形成一批产业集聚度高的中心镇和专业镇。培育发展一批中国名牌产品，形成汕尾名牌产业集群，走出一条“深圳研发、汕尾制造”、“深圳创造、汕尾配套”的产业发展之路。

　　——加强自主创新工作。全面实施创新驱动发展战略，借助深圳智力帮扶，推进汕尾市省级高新技术开发区、陆丰民营科技园区、陆河新河民营科技工业园等科技创新载体建设。加快海洋产业研究院、科创资本研究院、专业镇公共技术服务平台、检验检疫技术服务平台、汕尾广东水产品出口服务平台建设。深化“产学研”合作，增强企业自主创新能力。加强知识产权培育、保护和运用，激发全社会创新活力。推进质量强市活动。加大经济战略研究力度，推进环珠三角发展研究院建设。

　　（二）加快经济体制改革步伐，增强发展动力

　　抓住处理好政府和市场关系这个核心，立足建设良好市场环境，立足区域市场竞争力的提升，更加积极、更加稳妥地推进重点领域、关键环节改革，使市场在资源配置中起决定性作用和更好发挥政府作用。

　　——推动多种所有制经济共同发展。积极发展混合所有制经济，促进各类资本参与国有资本投资项目。积极推进企业去行政化和经营性资产统一监管。推动市属国有企业完善现代企业制度，构建科学合理的企业法人治理结构。以管资本为主加强国有资产监管。进一步促进民营经济的发展，鼓励乡贤回归、创业兴业。支持非公有制经济平等参与竞争，废除各种形式不合理规定，落实“非禁即入”投资政策。鼓励引导各类资本参与城市公共基础设施和社会事业建设，推进绿化造林等领域的市场化。

　　——建立公平开放透明的市场规则。推进金融、价格及要素市场改革，健全土地、资本、人力资源、技术、信息等要素市场。加快推进县城居民用水阶梯式水价改革，放开瓶装液化石油气销售价格。完善市场准入、运行、监管、退出制度和规范。推行商事登记改革，将“先证后照”改为“先照后证”，强化事中、事后监管，建立“宽进严管”的企业登记管理体制。推进社会信用体系和市场监管体系建设。加快建设统一的非银行信用信息共享平台。建设市公共资源交易平台。

　　——加快转变政府职能。启动新一轮政府职能转变和机构改革，全面梳理市场监管、农业农村管理、交通运输、城市规划建设和市政管理等部门职责，提高政府管理科学化水平。加快推进行政审批制度改革，制定公布审批事项目录并实行动态管理。规范和优化审批流程，建立“办事工作日”和“告知承诺”机制。稳步推进大部制改革。深化富县强镇、简政强镇事权改革，下移微观事权和执行权，促进政府管理扁平化。探索建立政府部门权责清单。推广政府购买社会服务，完善政府向社会转移职能制度。深化事业单位分类改革，推进法人治理结构、法定机构试点。

　　——深化财税金融改革。改进预算管理制度，建立全口径的预算编报体系，细化预算编制。加强专项资金规范管理。继续做好营业税改增值税试点。推动落实地方税体系建设，深化征管改革。优化金融生态环境，做大金融总量。积极引进各类金融机构、风投基金，设立投融资担保平台，扶持汕尾农商行发展壮大，支持信利光电等本地企业改制上市。完善保险服务体系，提高保险的深度和密度。

　　（三）加快产业园区建设，再掀招商引资热潮

　　坚持工业向园区集中、产业向园区集聚、项目向园区集结，坚持高起点规划、多元化融资、市场化运作，打造招商引资载体，推动产业园区扩能增效。

　　——加强园区分类指导。围绕高端装备、新材料、新能源等战略性新兴产业，通过改造升级、整合扩园、调整规划等方式，分步推进现有工业园区建设，构建一批主导产业突出的特色产业园区。争取上半年全市有3至4个产业园区获得省批准，纳入省级产业园区管理，享受省扶持政策。坚持错位发展、良性竞争，每个园区突出发展2至3个主导产业，实现产业集群发展。

　　——推进深汕特别合作区起步区建设。主动做好深圳市对口帮扶的对接工作，加快推进园区共建，尽快完成深汕特别合作区30平方公里起步区的规划、环评工作。拓展鹅埠信息产业组团，规划启动鲘门组团，建设小漠新能源基地，推进沿324国道的品牌产业带建设。加快组建合作区开发建设公司，推进园区污水处理、排水排污管网、起步区路网等基础设施建设。加快腾讯云计算数据中心、瑞和产业园等项目建设，推动华润大型云计算数据中心、深能源汕尾滨海电厂等项目尽快落地动工，形成合作区发展高潮。

　　——探索创新投融资机制。创新融资方式，搭建融资平台，成立园区管理开发机构，加强与国内投资机构、大型民企、省属国企以及商会、社团等对接合作，支持社会资本参与园区基础设施、配套服务设施和大型产业项目的投融资乃至整个园区开发建设。同时，采取BT、BOT以及产业基金等融资模式运作园区开发，尝试公开拍卖园区公用设施经营权、广告使用权等，努力实现由建设园区向经营园区转变。

　　——加大招商引资力度。以产业园区为载体，深入开展“招商引资”行动，全面铺开专业化招商，注重运用信息化、市场化手段，创新商业模式和合作模式，围绕主导产业，瞄准世界500强、中国500强、央企、省企、大型民企，有针对性地开展定向招商、专题招商和产业链招商，再次掀起招商引资热潮。注重打造战略合作平台，推动省市联合招商。充分利用对口帮扶政策，争取纳入深圳招商平台。充分发挥商会、行业协会和大型企业作用，大力推进“乡贤反哺工程”，争取在外汕籍企业家回乡投资发展。

　　——推动外贸提质增量。稳定出口，扩大进口，加快推进口岸设施建设和通关便利化，加快培育外贸转型升级示范基地，鼓励引导优势产业做长产业链，培育出口名牌和优势企业，推进外经贸转型发展。大力实施“走出去”战略，加强同东盟国家在珠宝、海洋渔业等产业的合作，加快发展服务贸易和服务外包，开拓境外市场，努力发展直接贸易。

　　（四）加快新区规划建设，推动中心城区扩容提质

　　坚持规划先行、项目带动、产城融合，加快新区规划建设，推动中心城区扩容提质，走新型城镇化道路。

　　——加快新区规划建设。尽快组建新区协调机构，加快编制新区产业发展、生态环境、基础设施建设等专项规划，启动中央商务区、红草高新技术产业园区、遮浪半岛滨海度假区和金町湾滨海旅游区等起步区的规划建设，重点抓好主要功能建筑、主要公共服务设施、重大基础设施建设，推动滨海旅游商贸、海洋新兴产业等快速发展，形成“一城、两园、三区”功能格局，打造“沿海岸、链条状、组团式”的现代化滨海新城。

　　——加快交通等城市基础设施建设。全面实施《汕尾市综合交通规划》，启动市域快速路网建设，加快潮惠高速公路汕尾段、深汕高速长沙湾全互通出入口及潭西出入口等工程建设；推进市客运枢纽中心建设；做好广汕高速铁路、龙（川）汕（尾）铁路、兴宁至汕尾高速公路、沿海快速通道汕尾市区至陆丰段及海丰支线等项目前期工作。主动融入深圳港口规划，加快推进红海湾新港区10万吨级等一批深水泊位码头及航道建设。加快市生活垃圾无害化处理中心首期工程焚烧发电厂、火车站站前广场及周边道路、市区金湖路东段、新湖大道南段、金鹏路及文德路北段、工业大道西段等重点市政工程建设。

　　——提升中心城区品质。坚持新城建设和老城改造、城市扩容和中心城区提质并重。在老城改造中突出汕尾特色和文化元素，增加公共服务供给，打通市区一批断头路，完善市政道路、排水排污、园林路灯、沿路绿化、公共休闲场所等公共设施，推动城乡公交一体化。

　　——加强城市管理。创新城市管理体制，积极稳步推进综合行政执法改革，推动城市管理重心下移，进一步发挥市城区、街道主体作用，建立“网格化”管理平台。加强市容环境综合整治，全面推行市区“门前三包”责任制。加强户外广告管理、市政设施维护和园林绿化管养，加快LED路灯改造和推广应用进度，加强城市交通秩序管理，努力创建“省卫生城市”。

　　——加快推进新型城镇化。科学规划，进一步优化城镇化布局和形态，引导人口、产业和要素资源往城镇集聚，有序推进农业转移人口市民化。积极推进县城和中心镇提质扩容，完善城镇基础设施，提高城镇公共服务水平，增强城镇综合承载能力，打造一批各具特色的中心城镇。

　　——提高信息化水平。加快信息化基础设施建设，完善数字化城市管理信息系统。推进信息技术在政务、商务、民生等领域的广泛应用，建设智慧汕尾。推进信息化与工业化深度融合，支持企业加快生产自动化、管理智能化、营销网络化建设。构建农村综合信息服务体系。效措施加以解决。

（五）加快构建新型农业经营体系，推动农业现代化和农村发展

　　积极培育新型经营主体，加快农业现代化建设，深入推进农村改革发展，不断增强农业农村发展活力，力促农业增效、农民增收、农村稳定。

　　——加强农村公共事业建设。加快高标准基本农田建设，抓好“百里海堤爱海工程”、治洪治涝保安工程、农田水利工程、村村通自来水工程。实施公平灌区节水改造项目。加快陆丰“三甲”海堤、陆丰市村村通自来水示范县建设。启动幸福村居创建行动，推进名镇名村示范村建设，改善农村人居环境。

　　——加快发展现代农业。把保障粮食安全和农产品质量安全摆在更加突出的位置，落实种粮补贴和农机购置补贴、政策性农业保险等强农惠农政策。充分利用深圳人才、资本、管理经验及市场优势，促进两市农业产业化对接。继续抓好23个省级“菜篮子”基地、9个省级“菜篮子”培育基地和产粮大县建设，打造一批现代农业示范园区。抓好华侨管理区特色农业基地建设。加快培育农业龙头企业、专业大户、家庭农场等新型经营主体。加快现代渔业转型升级步伐，组建远洋船队，大力发展深外海捕捞和水产品深加工业，推进汕尾（马宫）现代海洋渔业产业园区建设，创建 “南国渔都”。

　　——扎实做好村级换届选举工作。认真贯彻落实省委省政府工作部署，严格依法按程序做好选举工作，努力实现“两个80%”的工作目标，确保全面完成村、社区“两委”换届选举工作任务。

　　——深化农村综合改革。构建新型农业经营体系，推进家庭经营、集体经营、合作经营、企业经营等共同发展的农业经营方式创新，构建农业社会化服务新机制。建立健全农村土地仲裁机构，加快农村土地经营权确权，依法有序推动农村集体土地经营权流转，逐步推进农业适度规模经营。加强农村集体“三资”管理。稳步推进海丰、陆河两个山区县农村综合改革。

　　（六）加快生态文明建设，推进绿色低碳发展

　　坚守生态保护底线，建立健全系统完善的生态文明体系，促进绿色、循环、低碳发展，努力建设人与自然和谐相处的美丽汕尾。

　　——切实保护生态环境。划定生态保护红线，加强对敏感地带及海岸线、湿地、森林、矿山等重要资源的保护和管理，严厉打击乱砍滥伐、盗采稀土和河砂等违法行为。严格环保准入和污染物排放标准。实施最严格的水资源管理制度，以严格有效措施抓好公平水库、龙潭水库和螺河等重要水源地的水资源保护。大力推进以品清湖为重点的“美丽港湾”建设。加快农村连片环境综合整治。加快市环境监测监控中心项目建设，完善环境监测预警体系。加快7个中心镇污水处理厂和医疗废物无害化处理中心等污染防治设施建设，加大大气、固体废物、重金属污染防治力度。加快建设陆河生态文明县。

　　——注重资源节约利用。加强海域规划和综合利用，修订海洋功能区划，推动节约集约用海。实施最严格的耕地保护制度，落实基本农田保护经济补偿制度。抓好土地利用总体规划落实，实施最严格的节约集约用地管理。创新土地征收储备机制，抓好闲置土地清理处置，力争实现市区新储备土地3000亩目标。改进征地管理，完善征地补偿措施，统筹考虑被征地农民的长期生计，把更多的利益留给基层群众。加快保障性住房建设和“三旧”改造。加强土地执法检查，坚决打击违法违规用地行为。

　　——开展新一轮绿化大行动。本级财政投入2000万元推进全市新一轮绿化大行动。大力推进生态景观林带、森林碳汇、森林进城围城、乡村绿化美化四大重点林业生态工程建设，完成400公里生态景观林带和21万亩森林碳汇造林工程建设任务，建成260个村头公园。加快重点森林公园、湿地公园建设。严格执行森林防火责任制，提升森林管护水平。

　　（七）加快发展民生事业，建设幸福汕尾

　　按照守住底线、突出重点、完善制度、引导舆论的思路，切实做好改善民生各项工作，让改革发展成果更多更公平惠及全市人民。

　　——合理提高底线民生保障。继续推进社保扩面征缴，完善社会保险关系转移接续政策，推进市级、城乡、普通门诊“三项统筹”。培育完善政府主导、商业保险参与的医保经办管理新模式，推动统筹城乡居民的多层次医疗保障体系建设。建立市县津补贴水平分级分档调整制度，健全企业工资决定和正常增长机制，形成合理有序的收入分配格局。健全社会救助管理，把五保供养标准提高至2013年度农村人均年收入60%的水平。

　　——切实做好就业工作。实施就业优先战略，认真抓好促进就业创业政策的落实，健全就业服务平台，促进高校毕业生、就业困难人员、残疾人等重点群体的就业。鼓励自主创业、自谋职业，推动以创业带动就业。建立培训机构、企业、村三方合作机制，全面实施技能提升计划，加大农村劳动力技能培训力度，促进农村劳动力转移就业。

　　——全力推进教育领域综合改革。加大教育创强实施力度，统筹推进各级各类教育均衡发展，巩固提升高中教育质量，大力发展职业教育，力争新增省教育强镇20个。继续推进教育信息化工程，推进公共服务平台建设、信息技术普及应用等工作。加强师资队伍培训，提高师德师风建设水平。建设1000套以上教师公租房，逐步解决教师住房困难问题。加快推进特殊教育学校建设。支持鼓励民办教育发展。

　　——推进文化事业发展。全面推进“广东省文明城市”创建工作，广泛开展群众性文明创建活动。完善公共文化服务设施，推进中国（汕尾）民间文化艺术馆、市图书馆扩建和县区数字影院等建设。提高文化产业在经济总量中的比重。促进文化遗产的保护、传承和发展，做好第一次全国可移动文物普查工作。推动文学艺术创作，加强对外文化交流。开展“一县一品”文化品牌创建工作。

　　——提升卫生计生等公共服务水平。继续深化医药卫生体制改革，推进公立医院改革，巩固基本药物制度，逐步健全城乡医疗卫生服务体系。深化与中信集团合作，鼓励引进社会资本投入，加快推进市直医疗机构的改革。完善全市医药卫生信息平台。实施农村妇女“两癌”筛查项目。加强食品药品监管。巩固殡改工作成果，全面治理乱埋乱葬。加快推进体育场馆设施建设，广泛开展全民健身运动。强化责任制落实，实施优生健康惠民工程，完善利益导向机制，推进人口均等化服务，着力稳定低生育水平，实现人口计生前移进位目标。

　　——深化社会管理创新。推进社会管理体制改革。培育发展和规范管理社会组织，支持、引导其参与社会公共服务管理。推进基层社会管理体制改革，完善民事民治管理机制，推进社会协同善治。

　　——抓好平安汕尾建设。开展“平安建设”行动，创新立体化动态治安防控体系建设，加强反走私工作，深入开展陆丰涉毒整治，深挖保护伞，依法严厉打击盗抢销机动车犯罪等各类刑事犯罪活动。落实安全生产“党政同责、一岗双责、齐抓共管”，坚决防范和遏制重特大事故发生。完善综治信访维稳体系，实行网上受理信访制度，及时处理好群众合理诉求。推进气象现代化建设。建立健全应急体系，提高应急管理工作水平。

　　——实施新一轮扶贫开发。坚持扶贫先“扶志”，积极培育贫困群众自我发展意识，鼓励群众增强脱贫致富的信心和主动性。坚持“输血”与“造血”相结合，统筹推进帮扶基础设施建设，大力引导贫困村、贫困户发展符合当地特色且具有“造血功能”的产业项目，加快脱贫致富步伐。加强协同配合，努力为深圳市对口扶贫创造良好环境。加大财政投入，激发全社会关心、支持、参与扶贫开发的热情，营造携手扶贫、共建和谐的良好氛围。

　　同时，注重解决民生突出问题，重点办好十件民生实事。

　　（八）加快作风转变，加强政府自身建设

　　按照“为民、务实、清廉”的要求，扎实开展群众路线教育实践活动，全面履行政府职能，创新行政管理方式，切实加强政府自身建设。

　　——加强法治政府建设。自觉接受人大的法律监督和政协的民主监督，自觉接受舆论监督和群众监督。创新行政决策机制，推进政府决策的科学化、民主化、法制化。完善行政执法程序，规范行政执法行为。加强执法公开、政务公开、行政复议和行政应诉工作，扎实开展依法行政考评，落实行政执法责任和过错追究，不断提高依法行政水平。

　　——加强服务型政府建设。加大涉企涉民部门利民便民措施的出台落实和宣传引导力度，着力提高政府服务水平。加强电子政务建设，完善市行政服务中心和网上办事大厅，深入推进信息资源整合共享，拓展网上受理、并联审批和一站式服务。加快“12345”投诉举报平台建设。完善网络问政平台，促进民意征集常态化。充分利用微信、微博等信息手段做好政府信息公开工作。

　　——加强干部队伍作风建设。严格执行中央“八项规定”，深入开展政务整治行动，全面推进政务环境建设。加强政府机关工作人员的廉洁从政教育和廉政文化建设。强化权力运行制约和监督，继续采取明查暗访等有效措施，全面推进廉政风险防控。进一步突出作风建设，严纠“四风”，大力整治“庸懒散奢”等不良风气。严格落实反腐倡廉和厉行节约各项规定，认真开展收送“红包”礼金、小金库、违规使用专项资金、公务用车、违规修建楼堂馆所和办公用房等专项清理，严格控制“三公”消费和一般性支出。

各位代表！今天的汕尾已经站在一个新的发展起点上，面对人民的新期盼，我们深感愿景美好、使命光荣、责任重大。让我们更加紧密地团结在以习近平同志为总书记的党中央周围，在市委的坚强领导下，进一步解放思想，改革创新，脚踏实地，真抓实干，为加快汕尾振兴发展而努力奋斗!
